# Practical Notes on Diagnosis and Treatment

**Published:** 1906-07-07

**Authors:** 


### Practical Notes on Diagnosis and Treatment.

**Acetozone.**

If acetozone is ordered in solid form it should always be mixed freely with sugar of milk and administered in a capsule. Given alone it is very likely to cause vomiting.

**Garlic in Bronchiectasis.**

The drug which has given the best results in bronchiectasis is garlic. It may be given chopped and mixed with beef-tea or in capsules. As much as 30 grains eight times a day have been given. A fluid extract (dose ½ to 2 fluid drachms) and a syrup (dose 1 to 4 fluid drachms) are prepared. Another preparation is the volatile oil (dose ½ minim thrice daily); this may be prescribed in capsules and taken after food.—*Dr. Stanley Box.*

**Thyroid Extract in Migraine.**

It is suggested that in certain cases migraine may depend upon thyroid insufficiency, and this view finds support in a series of cases recently reported to the Hospitals Medical Society of Paris. In seven instances the patients were cured by the administration of thyroid extract.—*Lancet*, June 2, 1906.

